# Evolution and major changes of the diagnosis and treatment protocol for COVID‐19 patients in China 2020–2023

**DOI:** 10.1002/hcs2.45

**Published:** 2023-05-17

**Authors:** You Wu, Xiaoru Feng, Mengchun Gong, Jinming Han, Yuanshi Jiao, Shenglong Li, Tong Li, Chen Shen, Huai‐Yu Wang, Xinyu Yu, Zeyu Zhang, Zhengdong Zhang, Yuanfei Zhao, Peng Zhou, Haibo Wang, Zongjiu Zhang

**Affiliations:** ^1^ Institute for Hospital Management Tsinghua University Beijing China; ^2^ School of Medicine Tsinghua University Beijing China; ^3^ Department of Health Policy and Management, Bloomberg School of Public Health Johns Hopkins University Baltimore Maryland USA; ^4^ Nanfang Hospital Southern Medical University Guangzhou China; ^5^ Department of Neurology, Xuanwu Hospital Capital Medical University Beijing China; ^6^ Digital Health China Technologies Beijing China; ^7^ Liaoning Cancer Hospital and Institute Cancer Hospital of China Medical University Shenyang China; ^8^ The Second Affiliated Hospital of Chongqing Medical University Chongqing China; ^9^ MRC Centre for Environment and Health, Department of Epidemiology and Biostatistics, School of Public Health Imperial College London London UK; ^10^ National Institute for Health Research Health Protection Research Unit in Chemical and Radiation Threats and Hazards Imperial College London London UK; ^11^ National Institute of Traditional Chinese Medicine Constitution and Preventive Treatment of Diseases Beijing University of Chinese Medicine Beijing China; ^12^ Department of Neurosurgery, Union Hospital, Tongji Medical College Huazhong University of Science and Technology Wuhan China; ^13^ Department of Orthopedics The First Affiliated Hospital of Chengdu Medical College Chengdu China; ^14^ Beijing Institute of Heart, Lung and Blood, Beijing Anzhen Hospital Capital Medical University Beijing China; ^15^ Department of Neurosurgery The Third Affiliated Hospital of Soochow University Changzhou China; ^16^ Clinical Trial Unit, The First Affiliated Hospital Sun Yat‐Sen University Guangzhou China

**Keywords:** COVID‐19, diagnosis, treatment, clinical guideline

## Abstract

Since the identification of the first case of pneumonia of unknown cause in 2019, the COVID‐19 pandemic has spread the globe for over 3 years. As the most populous country in the world, China's disease prevention policies and response plans concern the health of the country's 1.4 billion people and beyond. During the course of the pandemic, scientific research has been accumulated and given evidence‐based support to the official guidance of COVID‐19 management. The National Health Commission of China have compiled, published, and updated a total of 10 versions of the “Diagnosis and Treatment Protocol for COVID‐19 Patients” to better inform clinical practitioners and staff to effectively screen, diagnose, manage, treat, and care for cases of severe acute respiratory syndrome coronavirus 2 infection. This paper compares and summarizes each version of the protocol in terms of etiology and epidemiology, clinical manifestation and diagnosis, treatment and nursing, disease control and management, presenting detailed changes, additions, deletions, and refinement of the protocols.

**Abbreviations**
2019‐nCoV: 2019 novel coronavirus
CCDC: Chinese Center for Disease Control and Prevention
Ct: cycle threshold
E: viral envelope
ECMO: extracorporeal membrane pulmonary oxygenation
M: matrix protein
N: nucleoprotein
NHC: National Commission of Health
S: spinosin
TCM: Traditional Chinese Medicine
WHO: World Health Organization

## 1 INTRODUCTION

**Figure 1:**
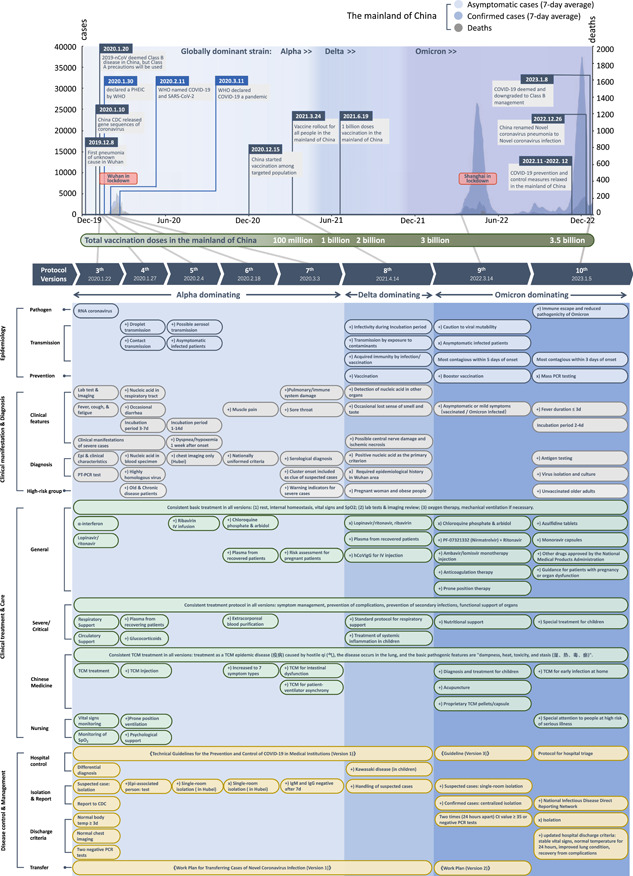
Timeline of major events of COVID‐19 and changes in the evolving versions of the diagnosis and treatment protocol for COVID‐19 patients in the mainland of China [11, 12, 13, 14, 15, 16, 17, 18]. CDC, Centers for Disease Control and Prevention; d, day; hCoVIgG, human COVID‐19 immunoglobulin (pH4); Ig, immunoglobulin; IV, Intravenous; PCR, Polymerase chain reaction; RNA, Ribonucleic acid; RT‐RCR, Real‐time polymerase chain reaction; SpO2, Saturation of Peripheral Oxygen; TCM, Traditional Chinese Medicine. “(+)” marks the items newly added in each version; “(−)” marks the specific items removed from the previous version; “(×)” marks the sections entirely removed from previous version.

On December 8, 2019 the first case of pneumonia of unknown cause was identified in Wuhan, China. The National Commission of Health of the People's Republic of China (NHC of P.R. China) immediately reported the emerging disease, referred to as the 2019 novel coronavirus (2019‐nCoV), to the World Health Organization (WHO), followed by rapid sharing of the genetic sequences for the nCoV by the Chinese Center for Disease Control and Prevention (CCDC) on January 11, 2020. It had been officially announced that 2019‐nCoV was deemed as a Class B disease on January 20, 2020, while Class A precautions would be used. WHO declared 2019‐nCoV a public health emergency of international concern on January 30, 2020 [1], and then officially named the disease as COVID‐19 [2]. COVID‐19 was declared by WHO as a pandemic later in March 2020 [3]. Nine months later, China started vaccination program among targeted population (staff engaged in import cold chain, port quarantine, ship pilotage, aviation aircrew, fresh market, public transportation, disease control, and other staff with a high risk of infection, and those who go to study or work in medium/high‐risk countries or regions) after the effective disease management and fast development of vaccine [4]. Vaccine rollout for the general population in the mainland of China was initiated on March 24, 2021 [5]. During the following 2 years (2021–2022), multiple variants for example, Alpha, Beta, Gamma, Delta, and Omicron have been evolved, with Omicron dominating the world. To date, the world has cumulatively seen over 670 million cases and 6.8 million deaths, while the number of confirmed cases has just reached 2 million in the mainland of China [6]. After 3 years of battle against COVID‐19, the prevention and control measures in the mainland of China was eased in November 2022 [7, 8]. In January 2023, the level of precaution for COVID‐19 has been downgraded to be managed as Class B disease in the mainland of China [9]. These major events in the context of the number of cases/deaths [10] in the mainland of China over the course of the pandemic are illustrated in Figure 1 (upper panel).

To better inform clinical practitioners and staff to effectively screen, diagnose, manage, treat, and care for cases of severe acute respiratory syndrome coronavirus 2 (SARS‐CoV‐2) infection, the NHC of P.R. China organized relevant experts to compile and publish a total of 10 versions of the “Diagnosis and Treatment Protocol for COVID‐19 Patients” (hereafter referred to as *the protocols*) [11, 12, 13, 14, 15, 16, 17, 18]^1^. We compared and summarized each version of the protocol in terms of four main aspects: (1) etiology and epidemiology, (2) clinical manifestation and diagnosis, (3) treatment and nursing, and (4) disease control and management. This review aims to show how national clinical guidelines have been updated and changed timely in the past 3 years to reflect the evolution of the disease and the scientific evidence accumulated over time (Figure 1, bottom panel).

## 2 ETIOLOGY AND EPIDEMIOLOGY

The evolving description of the pathogenesis, epidemiology, and prevention guidelines of COVID‐19 in each version of the protocol are summarized in Table 1.

**Table 1:** Major changes in pathogenesis, epidemiology, and prevention in the COVID‐19 clinical treatment protocols [11, 12, 13, 14, 15, 16, 17, 18].

Protocol versions	3th	4th	5th	6th	7th	8th	9th	10th
Date issued	2020‐1‐22	2020‐1‐27	2020‐2‐4	2020‐2‐18	2020‐3‐3	2021‐4‐14	2022‐3‐14	2023‐1‐5
Pathogenesis	A single‐stranded, positive‐sense RNA nCoV, belonging to the beta genus of coronaviruses Coronavirus is sensitive to heat and a variety of disinfectants can effectively inactivate the virus, while chlorhexidine cannot	(+) 85% homology with bat SARS‐like coronavirus (bat‐SL‐CoVZC45) (+) Coronavirus is sensitive to ultraviolet rays				(+) 2019‐nCoV has five essential genes respectively targeting RdRp and four structural proteins of nucleoprotein (N), envelope protein (E), matrix protein (M), and spike protein (S) (+) The S protein enters the cell by binding to ACE‐2	(+) 2019‐nCoV also named SARS‐CoV‐2 (+) 2019‐nCoV mutates and there are 5 VOC, namely Alpha, Beta, Gamma, Delta, and Omicron (+) The Omicron variant has replaced the Delta variant to become the dominant variant, and it is more transmissible than the Delta variant, but with weakened pathogenicity	(+) Genomic ORF (+) The recombination and mutation during the transmission may affect the biological characteristics of the 2019‐nCoV, leading to breakthrough infection and reinfection (+) Omicron variant demonstrates weakened lung pathogenicity, and the clinical presentation has changed from mainly pneumonia to mainly upper respiratory tract infections
Epidemiology		(+) Droplet and contact transmission (+) Universal susceptibility (+) Elderly and patients with underlying diseases are seriously ill (+) Children and infants are also affected (+) Epidemiological characteristics	(+) Asymptomatic infected patients (+) Possible aerosol transmission	(+) Transmission through close contact	(+) Aerosol or contact transmission caused by excrement to environmental pollution	(+) Incubation period infectivity (+) Transmission by exposure to contaminants (+) Acquired immunity by infection/vaccination	(+) Most contagious within 5 days of onset (−) Asymptomatic infected patients	(+) Most contagious within 3 days of onset (+) Vaccination benefits for the elderly
Prevention						(+) Vaccination (+) General precautions	(+) Booster shot	(−) Mass PCR testing (−) No longer identify the close contacts

Abbreviations: ACE‐2, angiotensin‐converting enzyme 2; CoV, coronavirus; ORF, open reading frame; PCR, polymerase chain reaction; RdRp, RNA‐dependent RNA polymerase; RNA, ribonucleic acid; SARS, severe acute respiratory syndrome coronavirus; VOC, variant of concern. “(+)” marks the items newly added in each version; “(−)” marks the items removed from previous version.

### 2.1 Pathogenesis

The subfamily of coronaviruses to which 2019‐nCoV belongs and its susceptibility to disinfectants remain unchanged in all versions of the protocol. With continuous exploration and research, the shape structure, and genetic characteristics of the pathogen have been described more specifically and precisely in each version. Since Version 8 of the protocol (April 14, 2021) [16], five essential genes of 2019‐nCoV correspond to nucleoprotein (N), viral envelope (E), matrix protein (M) and spinosin (S), and RNA polymerase (RdRp) were described in details. The infection process of the virus in the human body was also explained. The open read frame of 2019‐nCoV was provided in Version 10 of the protocol [18].

Mutations and recombination of 2019‐nCoV have led to the emergence of different variant strains, which in turn have implications for pathogenicity, detection and diagnosis, drug action, and also response to immunity acquired by infection and vaccine. The major strains of 2019‐nCoV, including Alpha, Beta, Gamma, Delta, and Omicron, have been described in the protocol Version 9 (March 14, 2022) [17], and it is emphasized that the variability of the virus may reduce the neutralizing effect of some monoclonal antibody drugs. Version 10 of the protocol (January 5, 2023) [18] described the subtypes of Omicron in more details, emphasizing that the pathogenicity of Omicron is significantly reduced and clinical presentations have been evolved from predominantly pneumonia to predominantly upper respiratory tract infections. However, the mutation can lead to a certain percentage of reinfection because it reduces the cross‐protection between different subbranch variants.

### 2.2 Epidemiology

The epidemiological characteristics of nCoVs have been added starting with Version 4 of the protocol (January 27, 2020) [12]. People infected with 2019‐nCoV (including those with asymptomatic infection) are the main source of infection, and transmission occurs through respiratory droplet transmission, contact transmission (including contact with contaminated objects), and aerosol transmission. The human population is generally susceptible to the virus, with the elderly and those with underlying health conditions being more severely affected. From Version 6 of the protocol (February 18, 2020) [14], the prerequisite of aerosol transmission (exposed to high concentrations of aerosols in relatively confined spaces) and the route of close contact transmission were added. Human excreta (feces and urine) can also transmit the disease, while mainly through aerosol or contact transmission by contamination. Version 8 (April 14, 2021) stated that individuals can also pass the virus during the incubation period. From Version 9 (March 14, 2022) to Version 10 (January 5, 2023), the timeframe of the highest infectivity was updated from within 5 days to within 3 days of disease onset due to the nature of the Omicron variant.

### 2.3 Prevention

From Version 8 (April 14, 2021) [16] onwards, the protocol included preventive measures for COVID‐19, mainly consisting of vaccination and general prevention measures. As evidence has shown that vaccination can provide immunity against the virus and reduce the risk of severe illness and death, all eligible persons are encouraged to get vaccinated. Those eligible for booster immunization should receive a booster shot. In Version 9 (March 14, 2022) [17], the second dose of booster immunization is promoted among those at high risk of infection, those aged 60 years and older, those with severe underlying disease, and those with compromised or suppressed immunity. General preventive measures include good personal and environmental hygiene. During the time when COVID‐19 was deemed Class B and managed as Class A disease (January 20, 2020–January 8, 2023), people with respiratory symptoms should seek medical attention at fever clinics. Those who have recently visited high‐risk areas or have a close contact with confirmed or suspected cases should get a polymerase chain reaction (PCR) test of COVID‐19 promptly.

Since January 2023, the NHC of P.R. China has downgraded the precaution level to manage COVID‐19 as a Class B disease. In Version 10 (January 5, 2023) [18] of the protocol, people infected with SARS‐CoV‐2 were allowed to be self‐isolated at home (instead of in quarantine facilities). Close contacts of confirmed cases will no longer be identified and PCR testing will no longer be mandatory.

## 3 CLINICAL MANIFESTATION AND DIAGNOSIS

The exams, pathology, symptoms, diagnostic criteria, and other clinical characteristics of COVID‐19 are summarized in Table 2. Major additions and modifications are extracted to show the improvement of the protocols over time. High‐risk populations and relevant warning indicators are also included.

**Table 2:** Major changes in pathology, clinical features, and diagnostic criteria in the COVID‐19 clinical treatment protocols [11, 12, 13, 14, 15, 16, 17, 18].

Protocol versions		3th	4th	5th	6th	7th	8th	9th	10th
Date issued		2020‐1‐22	2020‐1‐27	2020‐2‐4	2020‐2‐18	2020‐3‐3	2021‐4‐14	2022‐3‐14	2023‐1‐5
Pathology						(+) Pulmonary and immune system damage	(+) Detection of nucleic acid in other organs (+) Pathological changes do not include underlying disease lesions	(+) Description of lesion progression	(×) Removed
Clinical features	Lab test and imaging exam	Peripheral white blood cell and lymphocyte count, liver enzymes, muscle enzymes, myoglobin, CRP and ESR are abnormal Early chest imaging show multiple small patchy shadows and interstitial changes are seen, especially in the periphery of the lung. Then it develops into multiple ground glass shadows and infiltration shadows in both lungs	(+) The degree of lymphocyte is related to the severity of the disease			(+) Serological test	(+) Factors affecting specimen accuracy (+) Precautions for antibody diagnosis of 2019‐nCoV (+) Imaging manifestations of MIS‐C		
	Main symptoms	Fever, cough, fatigue, upper respiratory symptoms (few)	(+) Occasional diarrhea (few)		(+) Muscle pain	(+) Sore throat	(+) Occasional lost sense of smell and taste (+) Conjunctivitis (few)	(+) Asymptomatic or mild symptoms among those vaccinated or infected with the Omicron	(+) Most patients have low to moderate fever, but in some cases, patients can have high fever, which does not exceed 3 days (+) A small number of diseases develop to multiple organs and multiple symptoms
	Incubation period		Incubation period 3–7 days	Incubation period 1–14 days					Incubation period 2–4 days
	Severe cases	Acute respiratory distress syndrome, septic shock, metabolic acidosis, and dyscoagulation		(+) Dyspnea and/or hypoxemia 1 week after onset			(+) Possible (but rare) central nerve damage and ischemic necrosis of the extremities		(+) Severe patients often develop dyspnea and/or hypoxemia within 5–7 days after the onset
	Special population		Relatively mild symptoms in children				(+) Shortness of breath may be present in children and newborns (+) Possibly multisystem inflammation in children, prone to exacerbation		(+) Acute laryngitis, tracheitis and nervous system injury in children
Diagnostic criteria		Epidemiological history, clinical manifestations, RT‐PCR of viral nucleic acids, genetic sequencing of viruses	(+) Positive viral nucleic acid test on blood specimen (+) Highly homologous virus	(+) Confirmation by pulmonary imaging only in Hubei province	(+) Uniform diagnostic criteria inside and outside of Hubei Province	(+) Serological diagnosis (+) Cluster onset included as clue of suspected cases	(+) Diagnostic principles (+) Positive nucleic acid as the primary criterion (×) Removal of criteria from epidemiological history for Wuhan area (−) lgM, IgG increase more than four times		(−) Epidemiological history (−) Suspected cases (+) Antigen testing (+) IgG (+) Virus isolation and culture
Clinical classification			(+) Ordinary, severe, critical	(+) Mild	(+) Blood oxygen standard for severe cases at high altitude (+) Patients with lung imaging showing more than 50% of the lesion progress within 24–48 h are treated as severe cases	(+) Severe criteria for children	(+) Criteria for severe case in adults “Progressive aggravation of clinical symptoms” (+) Criteria for severe case in children “high fever duration ≥3 days”		Mild, moderate, severe, critical
Warning indicators						(+) Clinical warning indicators for severe/critical cases	(+) Modified clinical warning indicators for severe/critical cases		(+) Modified severe/critical clinical warning indicators for children
High‐risk population			(+) Elderly and people with chronic underlying diseases				(+) Late‐term pregnancies, perinatal women and obese people (+) Severe/critical high‐risk population identification criteria		(+) Older adults not vaccinated

Abbreviations: CRP, C‐reactive protein; ESR, erythrocyte sedimentation rate; Ig, immunoglobulin; MIS‐C, multisystem inflammatory syndromes in children; PCR, polymerase chain reaction; RT‐RCR, real‐time polymerase chain reaction. “(+)” marks the items newly added in each version; “(−)” marks the specific items removed from the previous version; “(×)” marks the sections entirely removed from previous version.

### 3.1 Pathological changes

In February 2020, a preprint of the Report on Gross Anatomy of Deceased Cadavers with Novel Coronavirus Pneumonia was published [19]. Thereafter, Version 7 (March 3, 2020) [15] of the protocol described the pathological changes based on the findings of the autopsy, describing the damage to the lungs and immune system (e.g., cytokine storm). In Version 8 (April 14, 2021) [16], the detection of SARS‐CoV‐2 nucleic acid in other organs was mentioned. Version 9 (March 14, 2022) [17] had additional descriptions of lesion progression. Pathological changes were not mentioned in Version 10 (January 5, 2023) [18] of the protocol.

### 3.2 Clinical presentation

Fever, dry cough, and fatigue are the main clinical manifestations of COVID‐19, which remained consistent across all versions of the protocol. Since Version 4 (January 27, 2020) [12], other concomitant symptoms have been added, including upper respiratory symptoms (nasal congestion and runny nose in some patients). Diarrhea, a loss or change to sense of smell or taste, and muscle pain were observed in a few patients, mostly recovering after 1 week. In a small number of patients, the disease continues to progress and pneumonia‐related symptoms may appear. In severe cases, the disease progresses to respiratory distress syndrome, septic shock, uncorrectable metabolic acidosis, and coagulation dysfunction. The risk of developing severe disease is higher in the elderly, those with chronic underlying disease, late pregnancy and perinatal women, and obese populations than in the general population. Childhood cases have relatively mild symptoms. Version 8 (April 14, 2021) [16] mentioned that vaccinated individuals and those infected with the Omicron strain are predominantly asymptomatic and mildly ill.

The major updates in the clinical presentation are summarized in the following aspects. The clinical incubation period was shortened as stated in Version 10 compared to that in Version 4 [12], probably due to increased infectivity by virus mutation. Before Version 9 [17], respiratory distress or (and) decreased oxygen saturation usually occur after 1 week among the severe patients, while in Version 10 [18] such symptoms showed up earlier, suggesting that the disease progresses faster than that before the Omicron strain prevailed. In this case, clinicians need to closely monitor patients' condition changes. Version 8 (April 14, 2021) [16] stated that a small number of children may develop a multisystem inflammatory syndrome, and the disease is likely to worsen in the short term. It is also stated in Version 10 (January 5, 2023) [18] that a few children may develop acute laryngitis and laryngotracheitis, and very rarely, life‐threatening neurological complications such as encephalitis, conjunctivitis, encephalopathy, acute necrotizing encephalopathy, acute disseminated encephalomyelitis, and Guillain–Barré syndrome. Therefore, in pediatric cases, special attention should be paid to its neurological complications.

### 3.3 Laboratory and imaging tests

The basic laboratory and imaging tests are consistent across the protocols. Version 5 (February 4, 2020) [13] of the protocol mentioned that some critically ill patients may have elevated troponin, suggesting that cardiac function in critically ill patients should be closely monitored. In Version 6 (February 18, 2020) [14], it was suggested that severe and critically ill patients often have elevated inflammatory factors, indicating that more intense anti‐inflammatory therapy might be required.

### 3.4 Diagnostic criteria

In each version of the protocol, the diagnosis of a case needs to be combined with epidemiological history, clinical manifestations, laboratory tests, and other comprehensive analysis, and a positive PCR test for the new coronavirus is the primary criterion for confirming the diagnosis.

The diagnostic criteria have undergone the following important revisions: the 3rd–9th versions of the protocol [11, 12, 13, 14, 15, 16, 17] had the diagnostic criteria for suspected cases, which were removed from Version 10 of the protocol [18]. Special medical observation of suspected cases is no longer required in the mainland of China in 2023. For pathogenic testing, Version 10 mentioned positive antigen testing and positive virus culture isolation as criteria to confirm the diagnosis of COVID‐19. It was emphasized that positive rapid antigen testing is sufficient for case confirmation, while a negative result cannot assert a person free of COVID‐19.

### 3.5 High‐risk population of severe/critical illness and clinical warning indicators

The absolute number of critically ill patients is expected to increase significantly in the course of peak infections, which may overwhelm the healthcare system. It is therefore important to define the population at risk of critical illness and intervene during early disease stages. From the 3rd–10th version of the protocol [11, 12, 13, 14, 15, 16, 17, 18], there is no change in the definition of criteria for the diagnosis of critically ill cases (in combination with one of the following conditions: 1. Respiratory failure requiring mechanical ventilation; 2. Shock; 3. Other organ failure requiring intensive care unit [ICU] monitoring). In Version 6 of the protocol (February 18, 2020) [14], the diagnostic criteria for severe cases were updated for high altitude environment (over 1000 m above sea level); indications of progression in pulmonary imaging were also mentioned. In Version 7 (March 3, 2020) [15], the diagnostic criteria for severe cases in children were refined, and additional clinical warning indicators for both adult and pediatric severe/critical cases were included. Version 8 (April 14, 2021) [16] further modified the details, such as progressive exacerbation of clinical symptoms; persistent high fever for more than 3 days was added as a diagnostic criterium for severe cases in children.

## 4 TREATMENT AND NURSING CARE

Across the versions of the protocol, the most significant modifications in the medical treatment, critical care, Traditional Chinese Medicine (TCM), and nursing care are presented in Table 3.

**Table 3:** Major changes in clinical treatment, Traditional Chinese Medicine, and nursing care in the COVID‐19 clinical treatment protocols [11, 12, 13, 14, 15, 16, 17, 18].

Protocol versions	3th	4th	5th	6th	7th	8th	9th	10th
Date issued	2020‐1‐22	2020‐1‐27	2020‐2‐4	2020‐2‐18	2020‐3‐3	2021‐4‐14	2022‐3‐14	2023‐1‐5
General treatment	Consistent basic treatment in each version: (1) rest, internal homeostasis, vital signs, and SpO_2_; (2) lab tests and imaging review; (3) oxygen therapy, mechanical ventilation if necessary							
	Alpha‐interferon nebulized inhalation Oral lopinavir/ritonavir Glucocorticoids	(−) Glucocorticoids	(+) Ribavirin IV infusion	(+) Oral chloroquine phosphate and Arbidol	(+) Maternal treatment precautions	(−) Lopinavir/ritonavir, ribavirin (+) Hydroxychloroquine and azithromycin are not recommended (+) Plasma from recovered patients (+) COVID‐19 human immunoglobulin (+) Glucocorticoids (+) hCoVIgG for IV injection	(+) PF‐07321332 (nirmatrelvir) + ritonavir (+) Ambavir/lomisvir monotherapy injection (+) Anticoagulation therapy (+) Prone position therapy (+) Psychotherapy (−) Chloroquine phosphate and Arbidol	(+) Azvudine tablets (+) Molnupiravir capsules (+) Guidance for patients with pregnancy or organ dysfunction (+) Other drugs approved by the NMPA
Critical care	Consistent treatment protocol in all versions: symptom management, prevention of complications, prevention of secondary infections, functional support of organs							
	Respiratory support Circulatory support	(+) Glucocorticoids (+) Gut microbiome (+) Psychological counseling (+) Plasma from recovering patients	(+) Oxygen inhalation (+) Salvage therapy (+) Extracorporeal blood purification	(+) Plasma from recovered patients	(+) Phlegm sucking (+) Bronchoscopy (+) Cycle monitoring (+) Renal replacement therapy (+) Tocilizumab (+) Risk assessment for pregnant patients (+) Intensive care of children	(+) Standard protocol for respiratory support (−) Concern for patients with sudden heart rate (+) Anticoagulant therapy (+) Treatment of systemic inflammation in children (+) ECMO therapy evaluation (+) Airway management	(+) Nutritional therapy	(+) Special treatment for children
TCM treatment	Consistent treatment in all versions: treatment as a TCM epidemic disease caused by hostile qi, the disease occurs in the lung, and the basic pathogenic features are “dampness, heat, toxicity and stasis”							
	Syndrome of novel coronavirus Pneumonia Confirmed Type‐specific TCM treatment protocols	(+) Stages of the disease (+) Staged Chinese patent medicine recommendation (+) TCM injection for medium and critical‐stage patients		Change “stage” to “classification” (unified with Western medicine) (+) Increased to 7 symptom types (+) Revised prescription (+) TCM Injection for severe and critical stage patients	(+) TCM treatment for intestinal dysfunction in mechanically ventilated patients (+) TCM treatment for patient‐ventilator asynchrony		(+) Ordinary cases‐plague poison and dryness syndrome (+) Diagnosis and treatment for children (+) Acupuncture (+) Proprietary TCM pellets/capsule	Change “Ordinary cases” to “Moderate cases” (−) Medical observation period (+) Mild, severe, and convalescent syndrome (+) Different types of syndrome differentiation and treatment plan for children, including external treatment plan (+) Chinese patent medicine recommendation (+) Severe and critical illness follow‐up treatment recommendations (+) TCM for early infection at home
Nursing care	Mentioned in the treatment protocols					Independent section		
	Rest in bed Vital signs monitoring Monitoring of SpO_2_	(+) Ventilation in prone position (+) Psychological support			(+) Closed suction	Monitoring of SpO_2_, ECG Mechanical ventilation (+) Early rehabilitation intervention		(−) Rest in bed, vital signs monitoring (+) Special attention to people at high risk of serious illness, especially oxygen saturation

Abbreviations: ECG, electrocardiogram; ECMO, extracorporeal membrane oxygenation; hCoVIgG, human COVID‐19 immunoglobulin (pH4); IV, intravenous; NMPA, National Medical Products Administration; SpO_2_, saturation of peripheral oxygen; TCM, Traditional Chinese Medicine. “(+)” marks the items newly added in each version; “(−)” marks the specific items removed from the previous version.

### 4.1 General treatment

The basic treatment in all versions of general treatment is essentially the same: (1) good rest in bed, supportive therapy, adequate energy intake; stability of the internal environment with water and electrolyte balance, vital signs, and finger oxygen saturation monitoring. (2) routine test of blood and urine, C‐reactive protein, biochemical indicators (liver enzymes, cardiac enzymes, renal function, etc.), coagulation function, arterial blood gas analysis when necessary, and chest imaging review. (3) Timely oxygen therapy according to changes of oxygen saturation, prone position ventilation for hypoxemia, transnasal high‐flow oxygen therapy, noninvasive or invasive mechanical ventilation, and anticoagulant therapy. Meanwhile, all versions have strict restrictions on the use of glucocorticoid and antibacterial drugs. Glucocorticoids can only be used when necessary, such as progressive aggravation of pneumonia and excessive activation of inflammatory responses. The dosage and duration should also be strictly controlled. Similarly, antibiotics can only be used if there is evidence of bacterial infection. Irrational or abuse of antibiotics is discouraged, especially the combination of broad‐spectrum antibacterial drugs.

The antiviral treatment regimens have been evolved over time. Version 3 of the protocol (January 22, 2020) [11] recommended inhaled interferon‐α nebulization [20] and lopinavir/ritonavir oral treatment [21]. Version 5 (February 4, 2020) [13] recommended ribavirin intravenous infusion treatment [22]. Version 6 (February 18, 2020) [14] recommended chloroquine phosphate [23] and arbidol oral treatment [24]. Meanwhile, the protocol outlined the instruction to care for patients with high‐risk factors of severe disease. Version 7 (March 3, 2020) [15] provided recommendations for pregnant individuals. Version 8 (April 14, 2021) [16] emphasized that antiviral agents should be used during an early disease course. For patients with rapid disease progression, human COVID‐19 immunoglobulin and tocilizumab [25] for intravenous injection were added as therapeutic options. Lopinavir/ritonavir, ribavirin, hydroxychloroquine, or a combination of azithromycin was no longer recommended. Version 9 (March 14, 2022) [17] recommended PF‐07321332 (nirmatrelvir) + ritonavir combination tablets [26], ambacizumab (BRII 196)/lomisvir (BRII 198) monoclonal antibody injection therapy [27]. Anticoagulation, prone position therapy, and psychotherapy were also added in Version 9 [17]. Version 10 [18] introduced Azvudine tablets [28], Molnupiravir capsule [29], and other drugs approved by the National Medical Products Administration for oral treatment of COVID‐19.

In terms of classified treatment, more attention has been paid to high‐risk groups and critical cases. Confirmed cases and critical cases were required to be admitted to designated hospitals and ICU, respectively, in Versions 3–8 [11, 12, 13, 14, 15, 16] Importantly, this had been updated and mild cases were not required to be transferred to designated hospitals and could be treated in centralized isolation in Version 9 [17]. Version 10 [18] emphasized that confirmed cases should be isolated according to the requirements of respiratory infectious diseases.

### 4.2 Critical care

The principles of treatment for critically ill patients remained the same in all versions: active prevention and treatment of complications based on symptomatic treatment, treatment of underlying diseases, prevention of secondary infections, and timely organ function support.

The main changes of respiratory support included: noninvasive mechanical ventilation for 2 h (the Version 3 and Version 4), transition to invasive mechanical ventilation if no significant improvement was noted, and invasive ventilation with a small tidal volume lung‐protective ventilation strategy to reduce lung injury. Prone ventilation, pulmonary resuscitation, or extracorporeal membrane pulmonary oxygenation (ECMO) was also included. The procedure of ventilation was introduced in detail in Version 5 (February 4, 2020) [13] of the protocol. Version 7 (March 3, 2020) [15] of the protocol set out the criteria for small tidal volumes and pulmonary rehabilitation therapy for invasive mechanical ventilation. The criteria and protocols for respiratory support were refined in Version 8 (April 14, 2021) [16].

For circulatory support, Version 7 (March 3, 2020) [15] of the protocol recommended monitoring of blood pressure, urine output, lactate and base residuals in blood gases, and pointed out the importance of observation for septic shock, gastrointestinal bleeding, or cardiac failure if there is a sudden increase in heart rate greater than 20% of the basal value or a decrease in blood pressure of approximately 20% or more of the basal value.

For other treatments, Versions 5 and 6 (February 4 and 18, 2020) [13, 14] mentioned blood purification therapy. Plasma therapy for recovered individuals was proposed in Versions 6 and 7 (February 18 and March 3, 2020) [14, 15]. In Version 7 (March 3, 2020) [15], precaution for acute kidney injury, attention to maintaining water–electrolyte balance, and indications for continuous renal replacement therapy were suggested. Multidisciplinary assessment of pregnancy risk in pregnant patients with severe/critical illness, termination of pregnancy if necessary, and cesarean delivery as the first choice. The use of tocilizumab as an immunotherapy and its recommended dosage were mentioned (not reflected in the 8th–10th versions [16, 17, 18]). Only Version 8 (April 14, 2021) [16] of the protocol mentioned anticoagulation treatment and the principles for multisystemic inflammatory syndrome in children [30]. Gammaglobulin and methylprednisolone dosages were added to Version 9 (March 14, 2022) [17]. Nutritional support was added to the protocol, as evidence has accumulated that critically ill patients are at extremely high risk of malnutrition [31]. In Version 10 (January 5, 2023) [18], more specific treatments of conditions in children with COVID‐19 were added.

### 4.3 Traditional Chinese Medicine

The following treatment protocols regarding TCM were consistent in all versions: treat COVID‐19 as a TCM epidemic disease (疫病) caused by hostile qi (气), the disease occurs in the lung, and the basic pathogenic features are “dampness, heat, toxicity and stasis (湿、热、毒、瘀).” From the 3rd–10th Versions, the TCM pathogenic features are gradually refined to cover the entire disease course, and the corresponding prescriptions have been provided. At the same time, nonpharmacological therapies such as acupuncture and tui‐na (acupoint massage) were also recommended, and the treatment plan for children had been refined gradually.

As the Omicron variant prevails and the clinical experience accumulated, the advantageous role of TCM in the prevention and treatment of COVID‐19 infections has been further highlighted. Version 10 (January 5, 2023) of the protocol [18] refined the TCM treatment recommendations for children with infections, providing detailed pharmacotherapy and external treatment protocols of pediatric tui‐na, gua‐sha, and acupuncture for children. Meanwhile, follow‐up treatment recommendations were provided for severe and critical illnesses of different disease stages and clinical symptoms. For early infections in non‐high‐risk groups, the proprietary Chinese medicines or Chinese medicine protocols were recommended in the guidelines [32] for Chinese Medicine Intervention at Home for People with COVID‐19 Infection could be followed. The guidelines recommended a variety of alternative proprietary Chinese medicines for adult and pediatric patients with different clinical manifestations, and provided treatment notes for special groups such as lactating women and elderly people with underlying diseases. Nonpharmacological Chinese medicine treatments, such as moxibustion therapy, meridian point massage, auricular point pressure, cupping, and Gong Fa exercises, were also recommended.

### 4.4 Nursing care

The nursing care of COVID‐19 patients is an important element of treatment and rehabilitation. Although it was not an independent section until the eighth version, the recommendations for nursing care had been gradually enriched with the accumulated understanding of the disease, the development of treatment methods and the application of new technologies. In Version 3 (January 22, 2020) of the protocol [11], the monitoring of patients' vital signs was mentioned, and in Version 4 (January 27, 2020) [12], the prone ventilation measures and psychological support for patients were added. Due to the formation of mucus and mucus plug in the pathological airway, nursing procedure of “closed suction according to the airway secretions” was added to Version 7 (March 3, 2020) [15].

Nursing care formed a separate section starting with Version 8 of the treatment protocol released on April 14, 2021 [16], and no major changes were made in the subsequent releases. It mainly included basic patient care, condition observation and monitoring (such as vital signs and oxygen saturation), intravenous access, position placement, and pressure sore prevention, treatment‐related care (such as care for noninvasive mechanical ventilation, invasive mechanical ventilation, artificial airway, prone ventilation, sedation and analgesia, ECMO treatment), oral care, fluid intake and output management, and psychological care. Due to the increasing frequency of noninvasive and invasive ventilation and the use of therapeutic measures such as artificial airway and ECMO, Version 8 (April 14, 2021) [16] started to include recommendations related to airway management, guiding operations such as airway wetting, aspiration, and sputum removal. Early rehabilitation was mentioned where lung recovery, physical rehabilitation, and psychological recovery were emphasized. In Version 9 (March 14, 2022) [17], the monitoring of vital signs was refined, focusing on ‘oxygen saturation at rest and after activity’. In Version 10 (January 5, 2023) [18], only the monitoring of vital signs, especially oxygen saturation at rest and after activity, was mentioned for high‐risk groups with serious illness.

## 5 DISEASE CONTROL AND MANAGEMENT

Table 4 lists the guideline of disease management and progression over time: nosocomial infection, differential diagnosis, case identification and reporting, isolation and hospital discharge, and patient transfer.

**Table 4:** Major changes in disease control and management in the COVID‐19 clinical treatment protocols [11, 12, 13, 14, 15, 16, 17, 18].

Protocol versions	3th	4th	5th	6th	7th	8th	9th	10th
Date issued	2020‐1‐22	2020‐1‐27	2020‐2‐4	2020‐2‐18	2020‐3‐4	2021‐4‐15	2022‐3‐14	2023‐1‐15
Hospital infection control	*Technical Guidelines for the Prevention and Control of COVID‐19 in Medical Institutions (Version 1)*						*Guideline (Version 3)*	Protocol for triage Medical staff management Medical waste management
Differential diagnosis	Other viral pneumonia Bacterial pneumonia Noninfectious diseases					(+) Kawasaki disease (in children)	(+) Close contacts: respiratory and pathogenic testing	(−) Close contacts: respiratory and pathogenic testing
Case identification and report	Suspected cases: isolation and treatment, pathogenic testing Report to CDC Two times negative nucleic acid tests can be excluded	Pathogenic testing of epidemiologically associated individuals	(+) Single‐room isolation (Hubei Province)	(−) Single‐room isolation (Hubei Province)	(+) IgM and IgG negative after 7 days can be released from isolation	(+) Handling of suspected cases	Suspected cases: single‐room isolation + two tests Confirmed cases: centralized isolation + reporting via direct network	Report through the National Infectious Disease Direct Reporting Network
Criteria for isolation release and hospital discharge	Normal body temperature ≥3 days Normal chest imaging Two negative PCR tests			(+) Precautions after discharge (+) Primary health care institutions follow‐up			Two times (24 h apart) *C*_t_ value ≥35 or negative PCR tests	(−) Isolation hospital discharge criteria: stable vital signs, normal temperature for 24 h, improved lung condition, no complications
Patient transfer	Transportation personnel protection Vehicle disinfection	*Work Plan for Transferring Cases of Novel Coronavirus Infection (Version 1)*					*Work plan (Version 2)*	(×) Removed

Abbreviations: CDC, Centers for Disease Control and Prevention; *C*_t_, cycle threshold; d, day(s); Ig, immunoglobulin; PCR, polymerase chain reaction. “(+)” marks the items newly added in each version; “(−)” marks the specific items removed from the previous version; “(×)” marks the sections entirely removed from previous version.

### 5.1 Hospital infection control

Versions 3–8 of the protocol [11, 12, 13, 14, 15, 16] referred to the Technical Guidelines for Prevention and Control of Novel Coronavirus Infections in Healthcare Facilities as the guidance for nosocomial hospital infection control. The first version of this guideline [33] provided detailed guidance on departmental management, medical staff protection, and patient management for all types of hospitals. In Version 9 (March 14, 2022) of the treatment protocol [17], hospital infection control was unified with the third version of the technical guidelines [34] to form a more systematic and standardized guidance. Recommendations for overall prevention and control strategies within and outside the healthcare facility have been added, while guidelines for the use of protective equipment have been eliminated. The latest Version 10 (January 5, 2023) of the protocol [18] reduced the reference technical guidelines by guiding prescreening and triage, medical personnel, and medical waste management.

### 5.2 Differential diagnosis, case finding, and reporting

The diagnosis of COVID‐19 infection in the treatment protocols is mainly differentiated from other known viral pneumonias (influenza virus, parainfluenza virus, adenovirus, respiratory syncytial virus, rhinovirus, human metapneumovirus, and SARS coronavirus), *Mycoplasma pneumoniae*, chlamydial pneumonia, and bacterial pneumonia. In addition, it has to be differentiated from noninfectious diseases, such as vasculitis, dermatomyositis and mechanized pneumonia. Since April 2020, medical personnel in United Kingdom, Italy, and United States have reported that some children infected by SARS‐CoV‐2 had symptoms similar to Kawasaki disease [35], and therefore a differential diagnosis needs to be considered. This point was added into Version 8 (April 14, 2021) [16] of the protocol. Version 9 (March 14, 2022) [17] of the protocol proposed that people in close contact with confirmed cases should be promptly tested for the pathogenesis even if they test positive for common respiratory pathogens.

Since the disease outbreak, countries around the world including China, responded quickly to establish terminology standards including new diagnostic codes, disease nomenclature, and test entries [36]. Cases of new coronavirus infection are always required to be reported after the disease has been included in Class B infectious diseases under the Prevention and Control of Infectious Diseases Law of the P.R. China and managed as a Class A infectious disease. Version 3 (January 22, 2020) of the protocol [11] instructed that upon discovery of a suspected case, medical personnel at all medical institutions should immediately isolate and treat the case and report it to the relevant department of the medical institution and the CCDC of the district. The medical institution should organize a consultation with relevant experts in the institution or district (county) within 2 h, and if the case cannot be diagnosed as viral pneumonia due to common respiratory pathogens, specimens should be collected promptly for pathogen testing. Suspected cases should be ruled out only if they have two consecutive negative nucleic acid tests for respiratory pathogens (at least 1 day apart). Version 4 (January 27, 2020) [12] included a recommendation for prompt pathogenic testing for those epidemiologically linked to COVID‐19 patients. Version 7 (March 3, 2020) [15] updated the diagnostic criteria to rule out suspected cases, that is, two consecutive negative nucleic acid tests (at least 24 h apart) and are still negative for coronavirus‐specific antibodies IgM and IgG 7 days after disease onset. In Version 9 (March 14, 2022) [17], for suspected cases or those with positive results of COVID‐19 antigen testing, their specimens should be collected immediately for nucleic acid testing or closed‐loop transfer to an upper‐level medical institution to perform nucleic acid testing, during which the persons should be subject to single person/single room isolation. If the nucleic acid test results were positive, the patient was subject to centralized isolation management or be sent to a designated hospital for treatment. Case information should be reported in accordance with the provisions of the network for direct reporting. In January 2023, COVID‐19 was downgraded to Class B management in the mainland of China. The reporting requirements have been relaxed in Version 10 (January 5, 2023) of the protocol [18], while the requirements for relevant reporting through the national direct reporting network for infectious diseases are listed.

### 5.3 Discharge criteria and transfer principles

The criteria for discharge from isolation and hospital in Version 3 (January 22, 2020) [11] were normal body temperature for more than 3 days, significantly improved respiratory symptoms, significant absorption of inflammation in lung imaging, and two consecutive negative PCR tests for respiratory pathogens (at least 1 day apart). In Version 6 (February 18, 2020) [14], postdischarge precautions for recovered patients during the concentrated outbreak of COVID‐19 infection was added, emphasizing the linkage between the designated hospital and the primary care institution, sharing medical records, and assisting patients with follow‐up visits after discharge.

Version 9 (March 14, 2022) of the protocol [17] included quantitative testing criteria for genetic testing methods, and the criteria for release from isolation were updated to two consecutive nucleic acid tests with cycle threshold (*C*_t_) values ≥35 for both N and open reading frame (ORF) genes (for fluorometric PCR: a cut‐off value of 40 and a sampling interval of at least 24 h) or two consecutive negative nucleic acid tests (for fluorometric PCR: a cut‐off value below 35 and a sampling interval of at least 24 h). The hospital discharge criteria were expanded to two consecutive nucleic acid tests with *C*_t_ values ≥35 for both N and ORF genes (for fluorometric PCR, a cut‐off value of 40 and a sampling interval of at least 24 h), or two consecutive negative nucleic acid tests (for fluorometric PCR, a cut‐off value below 35 and a sampling interval of at least 24 h). The isolation requirement was eliminated after COVID‐19 became a “Class B” infectious disease, and nucleic acid testing for inpatient discharge was no longer required in Version 10 (January 5, 2023) of the protocol [18]. At the same time, the clinician will make a comprehensive assessment of the infected patient's disease condition and recovery according to the conventional disease diagnosis and treatment requirements. When the disease condition has been improved significantly (the vital signs are stable, the body temperature is normal for more than 24 h, the lung imaging shows significant improvement of the acute exudative lesion), the patient can be switched into the oral medication. There are no complications that require further management. Instead, the discharge can be considered after an inquest by the physician.

The principles of transporting COVID‐19 patients have been formed based on the “Work Plan for the Transport of New Coronavirus Infected Patients” promulgated by China's State Council [37, 38]. Since Version 3 (January 22, 2020) of the protocol [11], it has been stipulated that patient should be transported in special vehicles, and personal protection and vehicle disinfection of transporters should be implemented. Transport‐related instructions were removed from the Version 10 (January 5, 2023) of the protocol [18].

## 6 CONCLUSIONS

The official diagnosis and treatment protocol for COVID‐19 reflected the authoritative recommendations of China's health administration and the relevant experts in the field. It has been guiding the clinical staff and the response teams to understand the disease, protect the population, and care for the patients. The major changes over the course of the pandemic are scientific‐supported and evidence‐based; these changes documented the evolution of the disease, the fast‐changing situation, and the adaptive healthcare management and response to COVID‐19 in China.

## AUTHOR CONTRIBUTIONS

**You Wu**: Visualization (equal); writing—original draft (lead). **Xiaoru Feng**: Visualization (equal); writing—review and editing (equal). **Mengchun Gong**: Writing—original draft (equal); writing—review and editing (supporting). **Jinming Han**: Writing—original draft (equal); writing—review and editing (equal). **Yuanshi Jiao**: Writing—original draft (equal); writing—review and editing (supporting). **Shenglong Li**: Writing—original draft (equal); writing—review and editing (supporting). **Tong Li**: Writing—original draft (equal); writing—review and editing (supporting). **Chen Shen**: Writing—original draft (equal); writing—review and editing (equal). **Huai‐Yu Wang**: Writing—original draft (equal); writing—review and editing (supporting). **Xinyu Yu**: Writing—original draft (equal); writing—review and editing (supporting). **Zeyu Zhang**: Writing—original draft (equal); writing—review and editing (supporting). **Zhengdong Zhang**: Writing—original draft (equal); writing—review and editing (supporting). **Yuanfei Zhao**: Writing—original draft (equal); writing—review and editing (supporting). **Peng Zhou**: Writing—original draft (equal); writing—review and editing (supporting). **Haibo Wang**: Conceptualization (lead). **Zongjiu Zhang**: Supervision (lead).

## CONFLICT OF INTEREST STATEMENT

The authors declare no conflict of interest.

## ETHICS STATEMENT

The authors have nothing to report.

## INFORMED CONSENT

The authors have nothing to report.

## Data Availability

The data of number of cases and deaths in the mainland of China from January 2020 to November 2022 are openly available at https://weekly.chinacdc.cn/news/TrackingtheEpidemic.htm.

## Footnotes

1 The first public release of the COVID‐19 diagnosis and treatment protocol was the trial version 3, which was published by the Chinese National Health Commission on January 22, 2020. Prior to this, the first and second trial versions were not made public.
